# Serosurvey of Bovine Viral Diarrhea Virus in Cattle in Southern Japan and Estimation of Its Transmissibility by Transient Infection in Nonvaccinated Cattle

**DOI:** 10.3390/v17010061

**Published:** 2025-01-02

**Authors:** Norikazu Isoda, Satoshi Sekiguchi, Chika Ryu, Kosuke Notsu, Maya Kobayashi, Karin Hamaguchi, Takahiro Hiono, Yuichi Ushitani, Yoshihiro Sakoda

**Affiliations:** 1 Laboratory of Microbiology, Department of Disease Control, Faculty of Veterinary Medicine, Hokkaido University, Kita 18, Nishi 9, Kita-Ku, Sapporo 060-0818, Hokkaido, Japan; 2 International Collaboration Unit, International Institute for Zoonosis Control, Hokkaido University, Kita 20, Nishi 10, Kita-Ku, Sapporo 001-0020, Hokkaido, Japan; 3 Hokkaido University Institute for Vaccine Research and Development (HU-IVReD), Hokkaido University, Sapporo 001-0021, Hokkaido, Japan; 4 One Health Research Center, Hokkaido University, Sapporo 060-0818, Hokkaido, Japan; 5 Center for Animal Disease Control, University of Miyazaki, Miyazaki 889-2192, Miyazaki, Japan; 6 Department of Veterinary Science, Faculty of Agriculture, University of Miyazaki, Miyazaki 889-2192, Miyazaki, Japan; 7 Kirishima Animal Consultation Clinic, Kirishima 899-5102, Kagoshima, Japan; 8 Japan Agricultural Cooperatives in Miyazaki Prefecture, Miyazaki 880-8556, Miyazaki, Japan

**Keywords:** age strata, bovine viral diarrhea, cattle, serological test, force of infection, basic reproduction number

## Abstract

Bovine viral diarrhea (BVD) is caused by the BVD virus (BVDV) and has been reported worldwide in cattle. To estimate BVDV circulation among cattle where few BVD cases were reported in southern Japan, 1910 serum samples collected from 35 cattle farms without a BVD outbreak were investigated to detect antibodies against BVDV-1 and BVDV-2 using an indicator virus with a cytopathogenic effect and the luciferase gene, respectively. Neutralizing antibodies against BVDV-1 and BVDV-2 were detected more frequently in 18 vaccinated farms than in 17 nonvaccinated farms. In the nonvaccinated farms, 9.6%, 1.8%, and 13.8% of the cattle were estimated to have a history of infection with BVDV-1, BVDV-2, and both, respectively. The median rate of within-herd anti-BVDV-1 seropositivity among cattle in the nonvaccinated farms was 22.0%; however, a high within-herd seropositivity (>50%) was confirmed in the two farms. The force of infection, basic reproduction number, and annual probability of BVDV-1 infection were estimated as 0.072 (95% confidence interval [CI]: 0.062–0.084), 0.36 (95% CI: 0.31–0.42), and 0.73% (95% CI: 0.61–0.87%), respectively, using the age-specific positive rate of anti-BVDV-1 antibodies. These parameters should be further applicable for developing epidemiological models which illustrate the BVDV dynamics in the field.

## 1. Introduction

Bovine viral diarrhea (BVD) is caused by the BVD virus (BVDV) and causes substantial economic losses to the livestock industry. It has multiple infection modes, including acute, chronic and persistent infection [1,2,3,4,5]. BVDV is divided into three genotypes, BVDV-1 (*Pestivirus bovis*), BVDV-2 (*P. tauri*), and HoBi-like Pestivirus (*P. brazilense*), belonging to the genus *Pestivirus* in the family Flaviviridae [6]. BVDV can spread horizontally via secretions, such as blood, nasal mucus, saliva, swabs, and urine [7,8,9,10]. Fetal transmission of BVDV can lead to abortion or induce fetal immune tolerance to BVDV, resulting in the delivery of calves with persistent infection (PI) [9]. Because such calves persistently shed infectious viruses, unless they are removed or die, they are considered primary sources of BVDV infection in herds [3,11].

The negative economic effects of BVDV infection are expected to be severe, prompting several countries to implement various control measures. Scandinavian countries have eradicated BVD by monitoring antibodies against BVDV and banning vaccinations [12,13]. These control measures are extremely stringent and require the culling of infected and recovered cattle. In contrast, Japan implemented the monitoring of PIs by detecting BVDV using reverse transcription-polymerase chain reaction or an antigen detection enzyme-linked immunosorbent assay kit and allowed vaccination as an optional measure. In Japan, BVD positivity is defined as the presence of PI cattle in both antigen detection tests with 2-week intervals [14]. BVD has been reported in Japan since the 1990s, and some control measures have been implemented. However, 0.12% of PI cattle prevalence (95% confidence interval [CI]: 0.05–0.25%) was reported in 2014 under the recommendation of BVD vaccination [14]. Because the prevalence of the three major subgenotypes of BVDV, including BVDV-1a, BVDV-1b, and BVDV-2a, was widely confirmed in Japan [15,16], vaccination against BVDV-1 and BVDV-2 has been strongly recommended. An effective vaccination program could reduce the risk of BVDV infection by increasing herd immunity and thus reduce the incidence of BVD cases; however, it cannot prevent the virus from circulating in the population. The number of BVD cases in Japan decreased from 265 in 2020 to 172 in 2023 [17]. Despite the decrease in cases, BVDV infections in these cattle appear to have reached an endemic state. To eliminate BVDV in Japan, a review of the current measures and identification of additional measures to minimize the incidence of BVDV infection should be considered. For example, vaccination against BVD is probably not constantly implemented, particularly in areas where BVD cases are rarely reported. This may be due to the lack of adequate knowledge among farmers about BVD and its systematic monitoring. Because PI calves continuously shed infectious viruses, the management of PI cattle on farms is the primary target of BVD control measures in Japan. Nevertheless, there has been an increasing concern regarding transient infection (TI)—also called acute infection—of BVDV in pregnant cattle. This condition is particularly significant in the context of implementing BVD elimination strategies as it can cause “trojan dam” in pregnant cattle between 25 and 90 days of gestation, although it may occasionally occur as early as 18 days or as late as 125 days [18,19]. However, TI cattle less frequently present clinical manifestations than PI cattle, and monitoring TI cattle in the herd is extremely difficult. Furthermore, a low prevalence of TI cattle may contribute to the poor efficacy of antigen monitoring.

Considering these epidemiological aspects, a serological survey of the cattle population, such as that implemented in Northern European countries for BVD elimination, should be an alternative method for understanding BVDV dynamics in the field. The results of such a serosurvey could provide epidemiological indicators of the target pathogen. The force of infection (FOI), defined as the rate of infection in a susceptible population, is a useful parameter for analyzing the dynamics of an infectious disease in a population and can be estimated using age-strata seroprevalence data. Several studies have estimated the FOI of a pathogen in a population by solving catalytic models and have also estimated the basic reproduction number (R_0_) of a pathogen, which represents the average number of secondary infections caused by a single primary and is used to estimate the occurrence and magnitude of a disease outbreak [20,21,22].

In the present study, serum samples of 2327 cattle were collected from 40 farms in Miyazaki Prefecture, southern Japan. Except sera from five farms where PI cattle were detected in the past 5 years, all 1910 serum samples from the remaining 35 farms were examined for antibodies to BVDV-1 and BVDV-2 using the serum neutralizing test (SNT). The seropositivity due to infections with field strains of BVDV in the study area was estimated using the SNT data of 892 cattle samples from 17 farms without BVDV vaccination. Moreover, the FOI was estimated through a serocatalytic SI model using age-strata seropositivity. In addition, R_0_ and the annual probability of infection by prevalent BVDV in the area were assumed.

## 2. Materials and Methods

### 2.1. Sample

In order to estimate sero-prevalence against BVDV-1 and BVDV-2 and the transmissibility of BVDV in the area where it was said that the vaccination against BVD had not been regularly implemented, all cattle farms in two cities (city A and B) in Miyazaki Prefecture, located in southern Japan, were targeted for the present study. Among the targeted farms, serum samples of 2327 cattle from 40 farms agreeing to participate in the present study were collected in 2023. Furthermore, general information on type of farm, including cattle introduction into the farm, use of calf development farms, application of the BVD vaccination, detection of PI cattle in the past 5 years, and the birth date of each cattle, was collected. Although two very small-scale farms (less than ten cattle in each farm) were included, most farms were small- or medium-scale in size, and they fed 15–177 cattle in each farm. Next, 381 serum samples from 5 farms with a history of PI cattle detection and 36 serum samples without the description of the host animal’s birth date were excluded from this study. Finally, 1910 serum samples from 35 farms (26 from city A and 9 from city B) were examined for the serological survey (Table 1). All the serum samples used in this study were obtained through the Japan Agricultural Cooperatives in Miyazaki Prefecture, with the cooperation of Miyazaki University. This study was approved by the ethical committee of Miyazaki University (approval number: 2023-025).

### 2.2. Neutralization Test Against BVDV-1 and BVDV-2

The serum samples were heat-inactivated at 56 °C for 30 min before the neutralizing test was performed. The SNT for BVDV-1 was conducted as described previously, with minor modifications [23,24]. Briefly, 50 μL samples of two-fold serial dilutions of sera were prepared using Eagle’s minimum essential medium containing 0.295% tryptose phosphate broth (Becton Dickinson, Franklin Lakes, NJ, USA) and 10% horse serum. These samples were prepared in 96-well microtiter plates and incubated with the same volume of 200 50% tissue culture infectious doses (TCID_50_) of a cytopathic BVDV-1 strain, the Nose strain, used as an indicator virus, for 1 h at room temperature [25]. Next, 5 × 10^3^ Madin-Darby bovine kidney (MDBK) cells were added to the mixture and incubated for 3 days at 37 °C in 5% CO_2_. The development of a cytopathogenic effect (CPE) in the well 3 days after inoculation was visualized by microscopy. The absence of CPE in the well was considered the presence of serum neutralizing antibodies against the indicator virus.

For the detection of antibodies to BVDV-2, recombinant BVDV-2 with high-affinity NanoLuc binary technology (HiBiT) luciferase gene was used as an indicator virus, and suppression of HiBiT detection was considered the presence of serum neutralizing antibodies against the indicator virus. Briefly, vBVDV-2 KZ-91-NCP/HiBiT was rescued from the recombinant full-length BVDV-2 KZ91-NCP strain inserted with the HiBiT luciferase gene at the middle of the viral E^rns^ region, which was kindly provided by Nippon Veterinary and Life Science University, Japan. Similar to the SNT to BVDV-1, the mixture of diluted serum, 200 TCID_50_ of vBVDV-2 KZ-91-NCP/HiBiT, and MDBK cells were incubated for 3 days at 37 °C in 5% CO_2_. The luciferase activity of the cell culture supernatant was measured using the Nano-Glo HiBiT lytic detection system (Promega, Madison, WI, USA) referring to a previous study [26]. The luciferase activity was measured using a 96-well LumiNunc^TM^ plate (Thermo Fisher Scientific, Waltham, MA, USA) installed in BioTek Synergy H1 (Agilent Technologies, Inc., Santa Clara, CA, USA). Neutralization of the indicator virus in each well was defined using the luciferase signal level lower than the cut-off of 20 luciferase activity, which was less than the average luciferase signal in mock-infected cells with five times their standard deviation. The serum neutralizing titers to either BVDV-1 or BVDV-2 of each serum sample were expressed as the reciprocal of the serum dilution at which the indicator virus was neutralized.

### 2.3. Estimation of Infection History with BVDV-1 and BVDV-2

A serum sample with an antibody titer of ≥4 was defined as a seropositive sample for the indicator virus [27]. For serum samples positive for both BVDV-1 and BVDV-2, when a higher SNT titer to one genotype was ≥4 times higher than the SNT titer to the other genotype, the sample was defined as being collected from cattle infected with the genotype with a higher SNT titer. In the previous experimental infection study, it was confirmed that the antibody with SNT titer of 512 against homologous strain was induced after the transient BVDV infection in healthy cattle [28]. Minami et al. demonstrated the 128 and 8 times of differences on SNT titers were recognized between Nose strains and BVDV-2 in antiserum against Nose-strain, and BVDV-1 and KZ-91 in antiserum against KZ-91 strain, respectively [29]. Given the geometric mean of these differences (32 times), SNT titer of cattle transiently infected with one BVDV genotype against the other genotype should be, at maximum, 16, implying that serum with >16 SNT titer against both genotypes cannot be simply explained as a non-specific reaction among heterologous genotypes of BVDV but may be the result of infections with both genotypes. For serum samples positive for both BVDV-1 and BVDV-2 but with SNT titers between BVDV-1 and BVDV-2 being <4 times or the SNT titer to both genotypes being ≥32, the sample was defined as being collected from cattle infected with both genotypes. For serum samples negative for both BVDV-1 and BVDV-2, the sample was defined as being collected from noninfected cattle.

### 2.4. Estimation of FOI and R_0_ Based on Seropositivity in Age Strata

The compartment model is frequently used to simulate disease transmission by monitoring the dynamics between each of the disease compartments. The basic model, also known as the SIR model, consists of three compartments, susceptible, infected, and immune/recovered. It is believed that in the absence of control measures, including vaccination, BVDV could circulate and persist in farms. A time-homogeneous model is suitable for accounting for the situation that individuals are added to a population without vaccination, or antibody responses due to exposure or infection to antigens under a disease endemic [30]. Under endemic equilibrium, there is no time dependence in the variables, resulting in the following set of ordinary differential equations [31]. The time-homogeneous model comprising three compartments is described as follows:

(1) $$\left\{\begin{array}{c} \frac{dS\left(a\right)}{da}=-\left(\lambda +\mu \left(a\right)\right)s\left(a\right)\\ \frac{dI\left(a\right)}{da}=\lambda s\left(a\right)-\left(\nu +\mu \left(a\right)\right)i\left(a\right)\\ \frac{dR\left(a\right)}{da}=\nu i\left(a\right)-\mu \left(a\right)r\left(a\right) \end{array}\right.$$

where *S*(*a*), *I*(*a*), and *R*(*a*) are the number of susceptible, infected, and recovered individuals at age *a*, respectively. The parameters of *λ* and *ν* are the FOI, which is the rate at which individuals are infected, and the recovery rate, which is often considered constant at any age. In general, the FOI should be affected by the age at which the cattle are exposed to the pathogen. The FOI was fitted to age-strata seropositivity by a serocatalytic SI model using the Rsero package in the R program version 4.2.1 (R Foundation), which performs Markov chain Monte Carlo simulation [32]. Among 12 different FOI scenarios set in the Rsero package, the best scenario model suitable for seropositivity in age strata was selected according to the deviance information criteria (DIC) obtained by running 5000 iterations with 4 chains in each of the 12 scenario models, and then its FOI was obtained. The constant of the FOI was used in this study under the assumption that all events occur with no time dependence, which is because of the lack of any association between susceptibility to BVDV infection and the age of cattle. The natural mortality rate at age *a* is denoted as *μ*(*a*). The survival function *δ*(*a*) is set as the probability of survival to age *a*, which is age-dependent, *δ*(*a*) = *e*^−*μa*^, using a constant mortality rate, *μ*, which equals a reciprocal of the life expectancy *L*. Under the assumption that the FOI is constant and not age-dependent, the average age at infection is equal to 1/*λ*. Using *L*, the R_0_ of the disease is given by *R* = *λL*. *L* was set as the average life expectancy of Japanese cattle.

## 3. Results

### 3.1. Detection of Antibodies Against BVDV-1 and BVDV-2 in Cattle

Among the 1910 serum samples collected from 35 farms, 892 and 1018 samples were derived from 17 farms without vaccination (from N1 to N17) and 18 farms with vaccination (from Y1 to Y18), respectively (Table 1). Because vaccination was implemented in the calf development farms when the cattle were introduced there, the farms transferring their cattle to calf development farms were also categorized into farms with vaccination. In cattle in the nonvaccinated farms, the rate of antibody positivity for BVDV-1 was 23.3%, which was similar to that for BVDV-2, whereas the rates of antibody positivity to each genotype in the vaccinated farms were 61.1% and 57.8%, respectively. Among the 17 nonvaccinated farms, the median within-herd seropositive rates for BVDV-1 and BVDV-2 were 22.0% and 18.6%, respectively. Each of them was significantly different from 75.0% and 70.5% in the vaccinated farms, respectively (*p* < 0.05). However, the antibody positivity rates for both BVDV-1 and BVDV-2 in two nonvaccinated farms were >50%. Regarding seropositivity at the farm level, only two farms (N5 and N11) were negative for both BVDV-1 and BVDV-2, indicating that herd seropositivity was 88.2%.

### 3.2. Examination of Cattle with a History of Infection with BVDV-1 and BVDV-2

**Figure 1:**
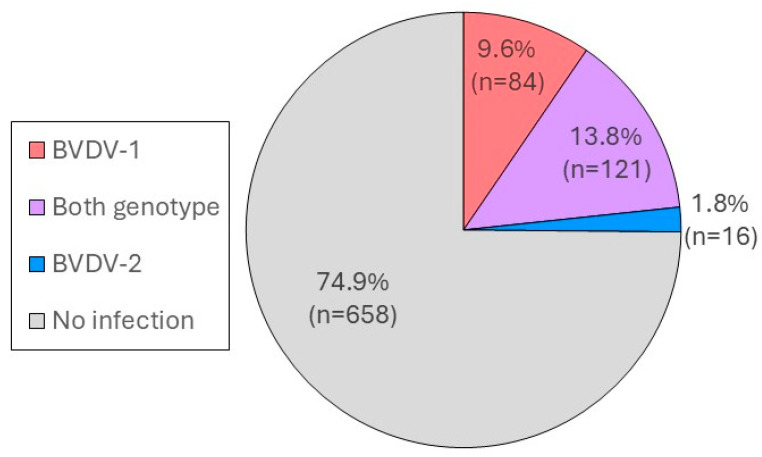
Estimated proportions of cattle infected with BVDV-1 and/or BVDV-2. All 892 cattle from 17 nonvaccinated farms were examined for seropositivity to BVDV-1 and BVDV-2. Cattle with SNT titers >4 to each BVDV genotype were defined as seropositive for the BVDV genotype. Cattle that were seropositive for both genotypes (BVDV-1 and BVDV-2) were considered infected with BVDV-1 if the SNT titer to BVDV-1 was four times greater than that to BVDV-2, and vice versa. Cattle were considered infected with both genotypes if the difference in the SNT titers between BVDV-1 and BVDV-2 were <4 times or in the SNT titers to BVDV-1 and BVDV-2 were ≥32. Cattle with SNT titers <4 to BVDV-1 or BVDV-2 were considered not infected.

Eight hundred and ninety-two cattle from 17 nonvaccinated farms (N1–N17) were examined for their history of BVDV-1 or/and BVDV-2 infection. Among 223 cattle samples with serum neutralizing titers >4 to BVDV-1 and/or BVDV-2, 85, 16, and 122 cattle were found to be infected with only BVDV-1, only BVDV-2, and both BVDV-1 and BVDV-2, respectively (Figure 1). Because the SNT titer to BVDV-1 was higher than that to BVDV-2 in the majority of cattle with antibodies to BVDV-2, these cattle were rarely categorized as being infected with only BVDV-2 and were hence categorized as being infected with only BVDV-1 or both genotypes. These findings emphasize that BVDV-1 rather than BVDV-2 had been sporadically circulating in the cattle population in this area. Using these findings, the epidemiological indicators were further estimated only for the majority in this study population, BVDV-1.

### 3.3. Seropositivity in Each Age Stratum and Estimation of FOI and R_0_

**Figure 2:**
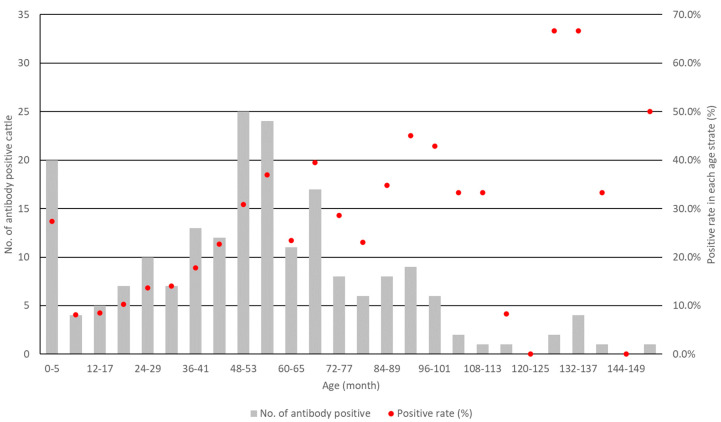
Numbers of cattle seropositive to BVDV-1 and its rates in each age stratum. Eight hundreds ninety-two cattle from 17 nonvaccinated farms were categorized into groups for every 6 months of age, and the rate of antibody positivity to BVDV-1 among the total animals in each age category (starting at 0–5 months) was calculated. Gray bar and red dot indicate the number of cattle seropositive to BVDV-1 and its rates, respectively, in the age strata.

Considering that there were no PI cattle detected in the 17 nonvaccinated farms in the past 5 years, the dynamics of BVDV-1 infection in this area were expressed using age-specific seropositivity. Cattle in the 17 nonvaccinated farms were categorized into groups for every 6 months of age, and the rates of antibody positivity to BVDV-1 among the total animals in each age category were calculated. Although seropositivity at age 0–5 months was high (27.4%), which probably indicated maternal immunity, seropositivity gradually increased in older age strata (Figure 2). Seropositivity reached approximately 40% in the age-stratum of 66–71 months and remained stable at that level in several age strata. Seropositivity at age higher than 114 months relatively fluctuated between 0% and 67%, probably due to the small sample size in those age strata.

**Figure 3:**
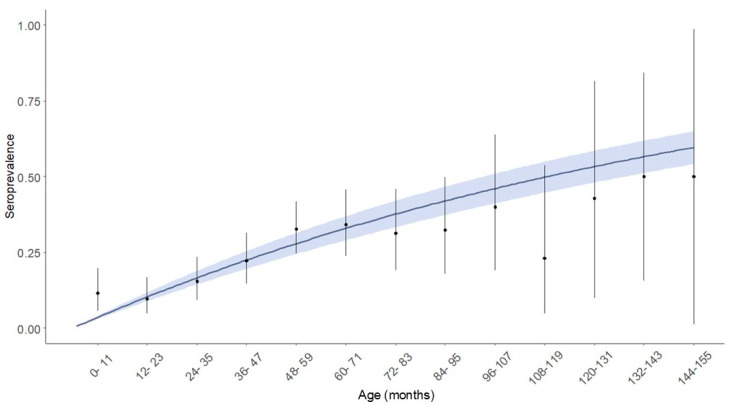
Age-specific seropositivity through the constant model with 95% confidence interval estimated based on seropositivity at each age stratum. Age-strata seropositivity is indicated by a curve solid line and fitted to estimate the force of infection in the constant model using a serocatalytic SI model using the Rsero package. The blue area indicates the 95% confidence interval estimated based on the same model.

To determine the best fit model among the 12 FOI scenarios in the age-strata SI model, the DIC of the 12 models using the age-strata seropositivity data were calculated. Because most DICs in the 12 scenarios were extremely similar at the lower level, the constant model, which indicates that seropositivity simply increases depending on the age-stratum, was adopted for further analysis. This approach could also be consistent with the fact that there were no interventions, including vaccination and culling, and no PI cattle detections. Based on the results obtained from the Rsero package, age-strata seropositivity to BVDV-1 in the constant model provided an FOI of 0.072 (95% CI: 0.062–0.084) (Figure 3) and an annual probability of infection of 0.73%. R_0_ was estimated as 0.36 (95% CI: 0.31–0.42) using the product of estimated FOI and the average life expectancy of Japanese cattle (5.5 years).

## 4. Discussion

This study examined a large number of cattle for seropositivity to BVDV-1 and BVDV-2. Among the cattle in farms that did not vaccinate and without PI cattle occurrence, one-fourth were estimated to be infected with BVDV, of which >20% had a history of infection with BVDV-1, whereas approximately 15% were infected with BVDV-2. These data are consistent with those of previous reports showing that BVDV-1, including BVDV-1a and BVDV-1b, was more prevalent than BVDV-2 in Japan [15,16]. This study also confirmed that higher seropositivity to BVDV-1 was proportional to age category, indicating that older cattle have greater exposure to field strains of BVDV.

Although seropositivity to both BVDV-1 and BVDV-2 was approximately 60% in vaccinated farms, it was approximately 23% in nonvaccinated farms at the animal level. This seropositivity rate in Japan is slightly lower than that in dairy farms with possible BVDV circulation (36.1%, 95% CI: 34.7–37.6%) but apparently lower than that in breeding beef cattle farms (54.5%, 95% CI: 48.5–60.5%) [29,33]. Moreover, this rate is similar to seropositivity to BVDV in African and South American countries, e.g., 18.24% in Egypt, 26.84% in Ethiopia, and 22.7% in Colombia [34,35,36]. A meta-analysis concluded that seropositivity to BVDV in nonvaccinated individuals at the global level was 45.19%, and, in Asia, it ranged from 41% to 51% [37]. The seropositivity observed in the present study may be reasonable or lower than that reported in the previous serosurveys. This discrepancy can be attributed to differences in cattle-feeding systems. Many farms in the area investigated in the present study were small-scale or backyard farms, and the seropositivity in the regions with large-scale commercialized farms could be high. Conversely, seropositivity to BVDV-1 in the nonvaccinated farms was 88.2% at the herd level, which was much higher than seropositivity to BVDV at the herd level in Ethiopia (68.3%) and the antibody positivity rates in bulk tank milk in Brazil (43%) and Poland (33%) [38,39]. Lower seropositivity at the animal level but higher seropositivity at the herd level may have originated from the unique husbandry system in Japan, which includes frequent animal movement from the market and between calf development farms. Furthermore, both animal- and herd-level seropositivity to BVDV-1 observed in the present study were higher than those reported in Ireland, where control measures against BVD were being implemented [40]. In addition to seropositivity, the detection of PI cattle in 5 of the 40 farms over the past 5 years implies that BVD risk persists in this area.

This study also aimed to simulate the dynamics of BVDV infection in nonvaccinated farms using a time-homogeneous SIR model. The FOI of BVDV could be estimated by solving a serological catalytic model describing the infection mode using serological data. FOI is a useful indicator for quantifying pathogen transmissibility and is used to calculate the R_0_ of an infectious disease, which is defined as the average number of secondary infections caused by a single typical case in a susceptible population. The transmissibility of transient BVDV infection should be much lower than that of PI. Thus, the transition of seropositivity is assumed not to correspond to age strata but instead leads to a dramatic increase at a threshold age, forming a sigmoid curve. Considering that no PI cattle had been reported in the farms investigated in this study and that seropositivity increased in an age-dependent manner in all nonvaccinated farms, the acquisition of antibodies against BVDV should be attributed to the continuous transient BVDV infection in the population. However, PI cattle may have been previously fed on two nonvaccinated farms with relatively higher within-herd seropositivity (N8 and N9). Several previous studies have estimated the epidemiological parameters of BVD dynamics. The R_0_s of BVDVs by TI were directly obtained by experimental infections and were calculated as 0.25 (95% CI: 0.01–1.95) for virulent BVDV-1 and 0.24 (95% CI: 0.01–2.11) for virulent BVDV-2 [41]. These values were not far from those reported in this study (0.36, 95% CI: 0.31–0.42). These data demonstrate that the R_0_ of transient BVDV infection did not vary according to the type of cattle and area and was certainly <1. The risk of “trojan dam” due to TI cannot be disregarded when animals are introduced into the herd. However, it is expected to be very low and unlikely to result in the constant birth of PI cattle. A relatively low R_0_ indicates the termination of BVDV infection within an infected individual rather than multiple infections to other individuals. Although the possibility of vertical infection from pregnant cattle to fetuses cannot be ruled out, TI cattle would rarely contribute to the spread of BVDV to other ones including pregnant ones, or produce PI cattle at the critical stages of gestation.

Initially, a serosurvey for BVDV was planned in populations where no BVDV vaccination programs had been implemented. This indicates that the focus on herd health in this area is likely lower than in areas where comprehensive herd health management measures, including vaccination and biosecurity, are effectively implemented. This might be a potential selection bias in the present study, and with an assumption that overall biosecurity measures in this area might be lower, the risk estimated through the present study could be higher than that in other areas. However, BVD vaccination is generally regularly implemented in most areas in Japan according to the recommendations. Furthermore, selecting a low-bias area was very difficult and eliminating the potential bias was nearly impossible. This is a limitation of the present study. Nevertheless, the risk estimated from the overestimated target population can indicate a very low risk of TI, providing important epidemiological insights for understanding BVDV dynamics in Japan.

For BVD control, although mass vaccination has been implemented in Japanese cattle herds for a long time, it has not been implemented in areas where the risk of BVDV was not prioritized. Serological investigation revealed that field strains of BVDVs sporadically infected immunologically naïve cattle herds; however, these invasions should not be critical and lead to the sustainable circulation of BVDV, although sporadic birth of PI cattle was confirmed. These data could reveal the dynamics of BVDV that were “masked” by mass vaccination control measures, offering insights into the epidemiology of PI cattle birth. Moreover, the epidemiological findings of this study can be applied to develop more accurate disease models to achieve BVD eradication.

## Figures and Tables

**Table 1:** Epidemiological characteristics of the cattle farms investigated in this study (N = 35).

Farm ID	Location	Cattle Introduction	BVD Test	Vaccination Status	Vaccination History	Total Animals	Positive Rate to BVDV-1	Positive Rate to BVDV-2
N1	City A	No	No	No	No	42	16.7%	16.7%
N2	City A	Partially	No	No	No	34	2.9%	2.9%
N3	City A	No	No	No	No	58	22.4%	24.1%
N4	City A	No	At birth	No	No	53	3.8%	1.9%
N5	City A	No	No	No	No	61	0.0%	0.0%
N6	City A	Partially	No	No	No	30	26.7%	23.3%
N7	City A	Partially	No	No	No	46	34.8%	32.6%
N8	City A	Yes	No	No	No	75	61.3%	56.0%
N9	City A	Partially	No	No	No	43	79.1%	69.8%
N10	City A	Partially	No	No	No	97	24.7%	24.7%
N11	City A	No	No	No	No	9	0.0%	0.0%
N12	City A	Yes	No	No	No	4	50.0%	50.0%
N13	City B	Yes	No	No	No	47	27.7%	21.3%
N14	City B	No	No	No	No	92	20.7%	18.5%
N15	City B	No	No	No	No	59	22.0%	18.6%
N16	City B	No	No	No	No	100	5.0%	3.0%
N17	City B	Yes	No	No	No	42	11.9%	9.5%
Y1	City A	Yes	At birth	Yes	At calf developent farm	66	87.9%	86.4%
Y2	City A	Yes	Before pasture	Yes	At calf developent farm	37	83.8%	75.7%
Y3	City A	Partially	At birth	Yes	At calf developent farm	16	75.0%	75.0%
Y4	City A	Partially	Before pasture	Yes	At calf developent farm	20	65.0%	65.0%
Y5	City A	No	Before pasture	Yes	At calf developent farm	15	80.0%	73.3%
Y6	City A	No	Before pasture	Yes	At calf developent farm	31	90.3%	87.1%
Y7	City A	Partially	Before pasture	Yes	At calf developent farm	42	90.5%	85.7%
Y8	City A	No	At birth	Yes	At calf developent farm	39	30.8%	30.8%
Y9	City B	No	Before pasture	Yes	At calf developent farm	74	25.7%	24.3%
Y10	City B	No	Before pasture	Yes	At calf developent farm	60	28.3%	21.7%
Y11	City B	No	Before pasture	Yes	At calf developent farm	44	36.4%	29.5%
Y12	City B	No	Before pasture	Yes	At calf developent farm	52	48.1%	50.0%
Y13	City A	Partially	Before pasture	Yes	Vaccinated in farm	68	76.5%	77.9%
Y14	City A	Partially	Before pasture	Yes	Vaccinated in farm	45	82.2%	77.8%
Y15	City A	Partially	Before pasture	Yes	Vaccinated in farm	104	58.7%	45.2%
Y16	City A	Partially	At birth	Yes	Vaccinated in farm	177	49.7%	44.1%
Y17	City A	Partially	Before pasture	Yes	Vaccinated in farm	44	81.8%	70.5%
Y18	City A	No	All	Yes	Vaccinated in farm	66	81.8%	78.8%

## Data Availability

Data are contained within the article.
